# Lifestyle behaviors and serum vitamin C in the Thai population in Bangkok Metropolitan

**DOI:** 10.17179/excli2018-1203

**Published:** 2018-05-16

**Authors:** Somchai Boonpangrak, Tanawut Tantimongcolwat, Lertyot Treeratanapiboon, Pairoj Leelahakul, Virapong Prachayasittikul

**Affiliations:** 1 Center for Research and Innovation, Faculty of Medical Technology, Mahidol University, Bangkok 73170, Thailand; 2 Department of Community Medical Technology, Faculty of Medical Technology, Mahidol University, Bangkok 73170, Thailand; 3 Department of Clinical Chemistry, Faculty of Medical Technology, Mahidol University, Bangkok 73170, Thailand; 4 Department of Clinical Microbiology and Applied Technology, Faculty of Medical Technology, Mahidol University, Bangkok 73170, Thailand

**Keywords:** serum vitamin C, vitamin C dietary intake, lifestyle behaviors, smokers, alcohol drinkers, outdoor workers

## Abstract

This study aimed to investigate the influence of lifestyle behaviors on the vitamin C levels in the circulating blood of the Thai population in Bangkok Metropolitan. The participants (n=250) included community workers (i.e., construction and business office workers) from the Bangkok Metropolitan, and the participants were placed in various behavior and lifestyle groups (Group I: reference; Group II: alcohol drinkers; Group III: outdoor workers; Group IV: smokers; and Group V: combined). The results showed that the lowest and highest vitamin C intakes were 7 and 27 mg/day in Groups IV and III, respectively. Group I (indoor workers free of smoking and drinking), had the highest total serum vitamin C level (39.7 µmol/L), while Group V (outdoor workers with smoking and drinking), had the lowest value (12.5 µmol/L). Furthermore, Group V had the highest prevalence (44 %) of total serum vitamin C deficiency (<11 µmol/L), while Group I had the lowest deficient indication (8 %). The vitamin C dietary intake and total serum levels were positively correlated in the reference group (Spearman's correlation=0.402, p < 0.05) but not in the other four groups. The significant adjusted odds ratio of inadequate total serum vitamin C (< 23 µmol/L) was 2.90 (CI: 1.15, 7.31) in Group IV and 3.73 (CI: 1.42, 9.81) in Group V. Moreover, the tendency to have an inadequate total serum vitamin C level was demonstrated in the following order: Group I < II < III < IV < V. Our results indicated that outdoor workers (Group III) and smokers (Group IV) had a greater likelihood of having a vitamin C deficiency than the reference group. A high percentage of deficiency was clearly observed among the outdoor workers with smoking and drinking behaviors (Group V).

## Introduction

Oxidative stress is caused by an imbalance between free radical generation and anti-oxidative protection systems in the human body (Persson et al., 2014[40]). Free radicals are highly reactive and destructive molecules generated by an electron transport chain (ETC), cytochrome P450, and other cellular and sub-cellular functions. They can also be produced by non-enzymatic and enzymatic reactions, such as Fenton's and Haber's reactions, and monoamine oxidase (Noori, 2012[36]; Orient et al., 2007[38]; Bedard and Krause, 2007[5]). Over production of free radicals leads to oxidative stress, which may create disturbances in body homeostasis and cause disease (Zampetaki et al., 2013[62]; Lastra et al., 2014[27]). Moreover, free radicals can also be generated from external sources (e.g., environmental pollution, cigarette smoke, alcohol, sunlight, and toxic metals) (Aseervatham et al., 2013[3]), which can also damage body systems. To maintain body homeostasis, both endogenous and exogenous antioxidant systems must be involved. Endogenous antioxidants are composed of enzymatic and non-enzymatic approaches. Superoxide dismutase (SOD), catalase, and glutathione peroxidase are an example of enzymatic antioxidants, while glutathione, ferritin, uric acid, lipoic acids, and coenzyme Q are non-enzymatic antioxidants (Poljsak et al., 2013[43]). Both antioxidant systems help protect our bodies from the dangerous effects of free radicals. For instance, SOD and catalase are used to catalyze superoxide anions and hydrogen peroxide, which are toxic substances, to non-toxic products (water). In addition, glutathione scavenges hydroxyl anions, hydrogen peroxide, and chlorinated oxidants (Sung et al., 2013[53]), while uric acid scavenges reactive free radicals from hemoglobin auto-oxidation and peroxide radical production from macrophages (Sautin and Johnson, 2008[47]; Ames et al., 1981[2]). Moreover, exogenous antioxidants from fruits and vegetables, such as vitamin C, vitamin E, phenolic compounds (e.g., phenolic acid, cinnamic acid), carotenoids (e.g., beta-carotene), and flavonoids (quercetin and rutin), demonstrate a synergistic effect on endogenous antioxidants as a total defense mechanism (Willett, 2006[59]). It has been suggested that consuming various exogenous antioxidants from food and dietary sources leads to a healthy life (Lopez and Denicola, 2013[31]). Among the exogenous antioxidants, vitamin C is an important vitamin that is involved in many anti-oxidative processes. 

Vitamin C (L-ascorbic acid) is an important water-soluble vitamin. It is primarily found in many fruits and vegetables (Du et al., 2012[15]). Due to a lack of gulonolactone oxidase (GLO) in the final step of vitamin C synthesis, humans, other primates and guinea pigs cannot synthesize this vitamin (Chatterjee et al., 1961[9]); hence, they depend on exogenous sources to maintain homeostasis, particularly from fruit and vegetables. Aside from its antiscorbutic property, vitamin C enhances immune functions, iron absorption, and collagen synthesis (Mandl et al., 2009[33]; Schlueter and Johnston, 2011[49]). Vitamin C also plays a role in the brain as a cofactor of dopamine beta-hydroxylase, which is involved in catecholamine synthesis (May, 2012[34]). As a free radical scavenger, vitamin C is the most effective external antioxidant in plasma due to its water solubility and ability to scavenge a wide range of reactive oxygen species (ROS) (Frei et al., 1990[19]), resulting in the protection of biomolecules from oxidative stress (Sung et al., 2013[53]; Du et al., 2012[15]; Descamps et al., 2001[13]). Vitamin C works synergistically with vitamin E to remove lipophilic radicals in lipid peroxidation (Du et al., 2012[15]; Kojo, 2004[26]). In humans, the vitamin C dose required to saturate plasma and tissues in healthy adults is 500 mg (Levine et al., 2001[29]), which is far from the recommended daily intake (60 mg) for disease prevention and general health promotion (Carr and Frei, 1999[8]; Deruelle and Baron, 2008[12]). However, higher doses (more than 500 mg) cannot change plasma levels due to the increased vitamin C excretion in urine (Levine et al., 1996[28]: Friedman et al., 1940[20]). Although a low amount (10 mg/day) of vitamin C is needed to prevent scurvy (Smith and Hodges, 1987[51]), a large cross-sectional study has demonstrated that vitamin C deficiency is commonly observed in humans and affects 5-10 % of adults in the industrialized world (Lindblad et al., 2013[30]).

Cigarette smoking and alcohol consumption have been associated with increased free radical production, leading to oxidative stress and antioxidant reduction (Wu and Cederbaum, 2013[60]; Valavanidis et al., 2009[55]). Schectman et al. (1989[48]) have reported that an inverse association between cigarette smoking and serum vitamin C level were independent of dietary intake. Furthermore, low blood levels of vitamin C and other antioxidants (vitamin A, C, E, and coenzyme Q10) have also been reported in smokers (Song et al., 2009[52]). Smoking, a lack of physical activity, and insufficient daily fruit consumption were all associated with vitamin C deficiency (Pincemail et al., 2011[42]). Regarding alcohol consumption, Zloch and Ginter (Zloch and Ginter, 1995[63]) have reported that moderate alcohol consumption in guinea pigs caused a decrease of vitamin C in tissue and body pool concentration when compared to a control group. In Thailand, a non-significant difference in the vitamin C dietary intake between smokers and non-smokers has been reported (Jitnarin et al., 2008[23], 2014[24]), and a decrease of blood levels of vitamin C has also been observed in priests or monks (Viroonudomphol et al., 2005[56]). In one survey, a 9.9 % blood vitamin C deficiency was observed in older Thai adults (Assantachai et al., 2005[4]). In the Bangkok Metropolitan, a large urban area, the lifestyle behaviors of cigarette smoking and alcohol consumption are still quite high. Much of the population works as laborer in the city, such as working under the hot tropical sunlight in the construction industry. The lifestyle behaviors and dietary intakes vary. Therefore, the present study aimed to explore the association between total serum vitamin C levels and vitamin C dietary intake and lifestyle behaviors that are believed to induce body oxidative stress (e.g., smoking, alcohol consumption, and working outdoors, particularly under the tropical sunlight) among people in the Bangkok Metropolitan. 

## Materials and Methods

### Participants

The present cross-sectional study was reviewed and approved by the ethics committee of Mahidol University prior to starting the project (COA no. MU-CIRB 2015/145.2411). Participants were drawn from workers at 10 workplaces (3 construction areas and 7 business offices), which were randomly selected from among 50 workplaces in Bangkok Metropolitan areas using a random sampling method. A preliminary standard questionnaire about background characteristics and lifestyles was used to screen the participants. The exclusion criteria were chronic diseases, regular medication, pregnant and/or lactating women, and the consumption of a vitamin C supplement. After screening, the participants were selected from each workplace using a simple random sampling. The following inclusion criteria were used for each group: Group I, the reference group (non-drinkers, non-smokers, and office workers); Group II (people who drink alcoholic beverages frequently, at least one glass of approx. 200 mL/day, three days/week); Group III (people who worked outdoors); Group IV (people who smoke frequently, at least one cigarette/day, three days/week); and Group V, the combination group (people who drink alcohol, at least one glass/day, approx. 200 mL, and smoke cigarettes, at least one cigarette/ day, three days/week and worked outdoors). A total of 250 participants enrolled in this study. The participants were informed of the objectives of the research project and further experiments before being given their informed consent form to read and sign. 

### Anthropometric and blood pressure measurements

For each participant, weight, height, and waist circumference (WC) were determined using a standard method. Weight circumference, as recommended by the World Health Organization and the International Diabetes Federation, was obtained by measuring between the superior border of the iliac crest and the inferior margin of the ribs (Wang et al., 2003[57]). Body mass index (BMI) was calculated as weight (kg) divided by height squared (m^2^). Blood pressure, including resting systolic and diastolic blood pressure levels, was also measured.

### Dietary assessment

On the day of blood collection, the single 24-hour dietary recall method was used for the dietary assessment of vitamin C intake and other nutrients. Next, a face-to-face interview was conducted by interviewers who were trained in this method. Quantities of food or portion size were estimated using a standard teaspoon, tablespoon, cup, and household utensils. Nutrient intakes were calculated for each volunteer using INMUCAL-nutrient software (version 3, 2015) from the Thai dietary database of the Institute of Nutrition, Mahidol University, and the vitamin C intake of each group was compared to the Thai dietary reference intake (Thai DRI) from the Department of Health, Ministry of Public Health of Thailand (Nutrition Division, Dietary Reference Intake for Thais, 2003[37]).

### Blood collection and preparation

After recruiting the participants, the appropriate date for blood collection was arranged. One day before the blood collection, the participants were asked to fast for 12 hours. Fasting blood samples were collected by venipuncture for glucose, lipid profiles, complete blood count (CBC), and vitamin C analysis. EDTA blood was used for a complete blood count, NaF blood was used for glucose analysis, and serum was used for lipid profiles and vitamin C testing. Samples were kept on ice and protected from light during transportation to the laboratory. For vitamin C analysis, the serum was separated (after clotting in a cool box for approximately 30 minutes) by centrifugation at 1,500 rpm for 10 minutes at 4 °C. Serum was aliquoted and preserved with an equal volume of 10 % meta-phosphoric acid (MPA) and 0.05 % ethylenediamine tetraacetic acid disodium salt (EDTA) and then stored at -80 °C immediately for further analysis. Biochemical tests (glucose, lipid profiles, CBC) were performed using routine clinical laboratory protocol of the same day of blood collection.

### Laboratory measurements

Glucose, lipid profiles and CBC were analyzed with a Hitachi Modular P800 Chemistry Analyzer and Coulter LH 780 at the Center of Medical Laboratory Services, Faculty of Medical Technology, Mahidol University. Serum vitamin C (ascorbic acid) and total serum vitamin C (ascorbic acid + dehydroascorbic acid) were measured through liquid chromatography mass spectrometry (LC-MS/MS) using a matrix match standard. The LC-MS/MS method was developed based on the method of Karlsen et al. in 2005[25], with some modifications. Briefly, for serum vitamin C, 100 µL of clear supernatant from the preserved sample was diluted with 400 µL of 1 % MPA (meta-phosphoric acid) and 0.05 % EDTA (ethylenediamine tetraacetic acid disodium salt). Then, it was centrifuged at 10,000 rpm for 5 minutes at 4 °C. The supernatant was filtered through a 0.22 μm filter and transferred to an amber vial to protect the sample from light and analyzed immediately. Regarding the total serum vitamin C, the same protocols were applied as mentioned above, except that a solution of 0.05 % EDTA, 1 % MPA, and 2.3 mM TCEP (tris(2-carboxyethyl) phosphine hydrochloride) was used for 30 min at room temperature for the extraction and reduction step. The dehydroascorbic acid (DHAA) concentration was determined by subtracting the concentration of the serum vitamin C from the total serum vitamin C content. Serum vitamin C was analyzed in triplicate for each sample. To minimize the variation of analysis, an internal quality control sample was used in each run. The intra- and inter-assay coefficients of the variation of serum vitamin C (performed three times) were 3.3-5.5 % and 5.6-7.3 %, respectively. The reference range of adequate serum vitamin C was 23-85 µmol/L and an inadequate level was <23 µmol/L. An inadequate level consisted of an insufficiency (11-22 µmol/L) and deficiency (<11 µmol/L) of serum vitamin C (Jacob, 1994[22]; Smith and Hodges, 1987[51]; Reuler et al., 1985[45]).

### Statistical analysis

Data analysis was performed using statistical analysis software for Windows, SPSS version 18. The descriptive statistics used in this study were the means and median. Normality testing was performed by the Shapiro-Wilk test. Analysis of variance (ANOVA) and the Kruskal Wallis test were used, depending on the normality of the data. A post hoc test (Tukey's Honestly Significant Difference test) was also used to detect significant difference between the pairwise. Logistic regression was applied to estimate the likelihood of being inadequate total serum vitamin C (< 23 µmol/L). The association between inadequate serum vitamin C and different lifestyle behaviors was measured by odds ratio (OR) and 95 % confidence interval. Moreover, multiple logistic regression was also used to adjust for the OR of the different lifestyle behaviors using the backward method. Spearman's rank correlation was calculated to measure the association between total serum vitamin C and vitamin C dietary intakes. The level of significance was set at 0.05.

See also the Supplementary data.

## Results

### Sociodemographic and anthropometric profiles of the participants

The sociodemographic and anthropometric profiles of the participants (59.6 % male and 40.4 % female) are presented in Table 1(Tab. 1). 

The participants ranged in age from 32.6-42.1 years. The marital status between single and others were equal (approximately 50 %). In terms of their educational status, approximately 69 % of the participants had an education level less than or equal to a diploma level, while 31 % were bachelor graduates or higher. A total of 64.4 % of the participants had monthly incomes of less than 15,000 Thai baht, and 35.6 % earned a higher income. Weight, height, and BMI were similar across all groups. In addition, the waist circumference for most of the groups was approximately 80 cm.

### Nutrient intakes of various lifestyle behavior groups

The nutrient intake of Thai adults in various groups was explored (Table 2(Tab. 2)). The macro-nutrient intake (carbohydrate, protein, fat), carbohydrate and fat intake were not significantly different between the groups. However, protein intake demonstrated a significant difference (p value < 0.05). The highest and lowest values of protein intake were 62 and 43 g/day for Groups III (outdoor workers) and IV (smokers), respectively. In addition, the average median energy intakes in all groups for males and females were approximately 69 % and 83 %, respectively, compared to the Thai DRI. 

Regarding vitamins, most fat and water-soluble vitamins were not significantly different, except for vitamin E (p value < 0.05). The lowest (median 0.4, IQR 1.6) and highest (median 1.7, IQR 3.5) vitamin E dietary intakes were in Groups V (combined) and I (reference), respectively. However, the highest median vitamin E value was less than the Thai DRI (approximately nine times less). For vitamin C, the main source of dietary intake was from fruits and vegetables, such as oranges, guava, papayas, tomatoes and cabbage. Group III (outdoor workers) had the highest vitamin C dietary intake, with a median value equal to 27 mg, while group IV (smokers) had the lowest vitamin C intake (7 mg). The results indicated that the daily intake of vitamin C for each group was not sufficient when compared to the Thai DRI. For minerals (calcium, magnesium, phosphorus, copper, iron selenium, and zinc), the intakes of most minerals in the various groups were lower than the Thai DRI by approximately 1.3-3 times, except for magnesium, which was approximately 20 times lower. A significant difference among the groups was observed for only four minerals (calcium, copper, iron, zinc), with a p value less than 0.05, while the other three minerals (magnesium, phosphorus, selenium) were not significantly different. All results demonstrated that the median intakes of energy, macro-, and micro-nutrients in the various groups were lower than the Thai DRI.

### Baseline glucose, lipid profiles, and complete blood counts

The biochemical profiles of glucose, lipids and hemato-parameters of the blood of individual participants were assayed. The results of each group were analyzed and are shown in Table 3(Tab. 3). There was no significant difference in the plasma glucose levels among the groups. For lipid profiles (total cholesterol, triglyceride, high density lipoprotein-cholesterol: HDL-C and low-density lipoprotein-cholesterol: LDL-C), the disparity among the group was significantly demonstrated only for total cholesterol and LDL-C, with a p value < 0.05, according to the Kruskal-Wallis test. Regarding the quality of erythrocytes and number of corpuscles (complete blood count: red blood cell count, white blood cell count, platelet count, hemoglobin, hematocrit, mean corpuscular volume [MCV], and red blood cell distribution width [RDW]), most parameters were similar among the groups. Nevertheless, a statistically significant difference was observed for hemoglobin and hematocrit by ANOVA (p value < 0.05). In addition, the post hoc test (Tukey's Honestly Significant Difference test) demonstrated that the mean value (13.3 g/dL) of hemoglobin in Group III (outdoor workers) was significantly different compared to the other four groups, with a p value < 0.05, while the hematocrit level (40.7 %) in Group III (outdoor workers) was significantly different only compared to Groups I (the reference group, II (drinkers) and IV (smokers) (p value < 0.05). However, most results demonstrated that the basic biochemical parameters in the various groups were similar and within the reference ranges.

### Serum ascorbic acid, dehydroascorbic acid, and total ascorbic acid in various lifestyle behavior groups

Three forms of vitamin C (ascorbic acid (AA), dehydroascorbic acid (DHAA), and total ascorbic acid (AA+DHAA) were analyzed in the blood of all participants (Table 4(Tab. 4)). Ascorbic acid and total ascorbic acid were directly measured, and the DHAA value was calculated from the difference between the AA and total ascorbic acid. For ascorbic acid, the lowest (7.9 µmol/L) and highest (32.9 µmol/L) serum vitamin C levels were in Groups V (combined) and I (reference), respectively (Table 4(Tab. 4)). The serum vitamin C levels were demonstrated in the following order: Group I (reference) > III (outdoor workers) > II (drinkers) > IV (smokers) > V (combined) (32.9 > 23.3 > 21.6 > 14.2 >7.9 µmol/L). Regarding total ascorbic acid, Group I (reference) had the highest total serum vitamin C level (39.7 µmol/L), and the value was approximately 1.4-3.2 times higher than the other groups. Total serum vitamin C levels were demonstrated in the following order: Group I (reference) > II (drinkers) > III (outdoor workers) > IV (smokers) > V (combined) (39.7 > 28.4 > 23.3 > 19.3 > 12.5 µmol/L). In addition, the DHAA level was ranged from 2.3-6.8 µmol/L. Interestingly, a statistically significant difference was observed among the groups for the three forms of serum vitamin C, with a p value less than 0.001. When focused on gender, the highest and lowest total serum vitamin C levels in males and females were in Groups I (reference) and V (combined), respectively. In addition, in most of the groups, the females had a higher total serum vitamin C level than the males (Table 5(Tab. 5)).

### Prevalence of adequacy, insufficiency, deficiency, and inadequacy of total serum vitamin C among various lifestyle behaviors

**Figure 1:**
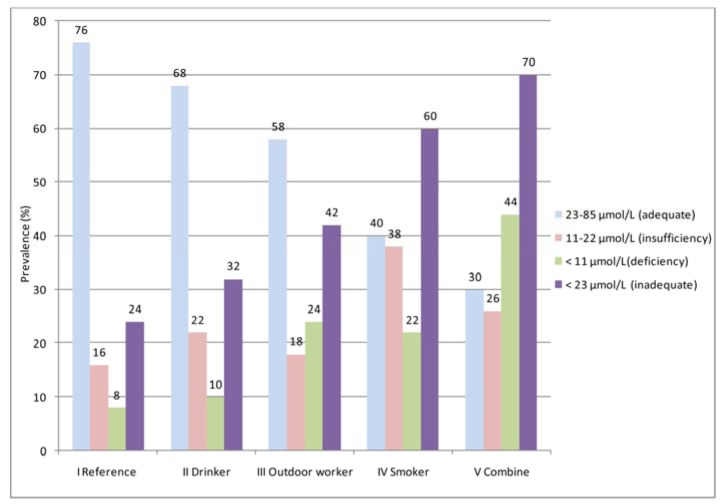
Status of serum vitamin C levels (adequate, insufficiency, deficiency, and inadequate) for the different lifestyle behaviors

The vitamin C status of the participants was graded as adequate (23-85 µmol/L), insufficiency (11-22 µmol/L), and deficiency (<11 µmol/L) (Figure 1(Fig. 1)). In Group I (reference), 76 % of the participants had adequate total serum vitamin C levels, while the four other groups (II to IV) were 68 %, 58 %, 40 % and 30 %, respectively. The highest vitamin C insufficiency (38 %) was found in Group IV (smokers), which was 1.5-2 times higher than the other groups. Vitamin C deficiency was also demonstrated, and the highest prevalence was found in Group V (combined) (44 %). This value was approximately five times higher than that of Group I (reference,8 %). Inadequacy (insufficiency + deficiency: <23 µmol/ L) of total serum vitamin C was also demonstrated. The trend of inadequate total serum vitamin C increased from Group I (reference) to Group V (combined). However, the observation was vice versa when focusing on adequate levels of this vitamin.

The OR and 95 % confidence intervals of inadequate total serum vitamin C (<23 µmol/ L) are shown in Table 6(Tab. 6). The odds ratio for the four groups (drinkers, smokers, outdoor workers, and combined) were calculated and compared to the reference group. The results showed that Group II (drinkers) was more likely to have an inadequate total serum vitamin C than the reference group (Group I), however, this difference was not statistically significant (OR=1.49, 95 % CI: 0.62, 3.59). Group III (outdoor workers) was approximately 2.3 times more likely to have an inadequate total serum vitamin C than Group I (reference) (95 % CI: 0.97, 5.41). An increased likelihood of having an inadequate total serum vitamin C was also pronounced in Groups IV (smokers) and V (combined). The odds ratios were 4.75 and 7.39, with significant differences for the former (p<0.01) and the latter (p<0.001) groups. After adjusting for gender and education, the odds ratio decreased. A significant odds ratio was still observed for both Group IV (smokers) (OR= 2.90, CI: 1.15, 7.31) and Group V (combined) (OR=3.73, CI: 1.42, 9.81). Regarding vitamin C dietary intake and the corresponding total serum vitamin C, the correlation between both parameters showed a positive association only for Group I (reference) (r=0.402, p < 0.05), according to Spearman's correlation (Table 7(Tab. 7)). 

## Discussion

### Serum vitamin C level among lifestyle behaviors

In this cross-sectional study, we found significant differences between the total serum vitamin C levels among the different lifestyle behavior groups. The baseline total serum vitamin C and dietary vitamin C intakes of Thai adults from the Bangkok Metropolitan area were analyzed among various lifestyle behaviors, including Group I, (reference), Group II (frequent drinkers), Group III (outdoor workers), Group IV (frequent smokers), and Group V (combined), people whose lifestyle behaviors included those of Groups II, III, and IV. As expected, the reference group (Group I) had a higher total serum vitamin C level than those of the other four groups. In comparison, the smoking group (Group IV) had an approximately two times lower total serum vitamin C level than the reference group. Our results support the notion that smoking is a risk factor for blood vitamin C reduction (Lykkesfeldt et al., 2000[32]; Dietrich et al., 2003[14]), which might be due to the high number of free radicals generated and the increased load of oxidative stress from cigarette smoking, resulting in a high turnover rate of ascorbic acid (Eiserich et al., 1995[16]; Pelletier, 1997[39]; Alberg, 2002[1]). A low consumption of a vitamin C-enriched diet might also be a cause (Pincemail et al., 2011[42]). 

For alcohol drinkers (Group II), the low serum vitamin C (1.4 times less than the reference group) may be primarily due to the diuretic property of alcohol leading to an increase in urinary ascorbate excretion. Potentiation of free radicals of ethanol metabolism might also be involved (Shohaimi et al., 2004[50]; Das and Vasudevan, 2007[10]; Faizallah et al., 1986[17]). This finding is supported by a decrease in body-pool vitamin C found after alcohol consumption (Zloch and Ginter, 1995[63]; Suresh et al., 1999[54]). 

Regarding the outdoor workers (Group III) with a low monthly income and daily hard work under sunlight exposure, our results confirmed a low serum vitamin C (Shohaimi et al., 2004[50]; Mosdøl et al., 2008[35]; Fletcher et al., 2008[18]; Bickers and Athar, 2006[6]; Péter et al., 2015[41]). Moreover, environmental factors, such as sunlight, UV light, xenobiotics, and air pollutants, which generate free radicals, may cause total serum vitamin C depletion. Note that a combination of lifestyle behaviors, including smoking, drinking, and heavy exposure to sunlight (Group V), intensifies the reduction of body vitamin C (Eiserich et al., 1995[16]; Shohaimi et al., 2004[50]; Das and Vasudevan, 2007[10]; Faizallah et al., 1986[17]; Mosdøl et al., 2008[35]; Fletcher et al., 2008[18]; Bickers and Athar, 2006[6]; Péter et al., 2015[41]). 

### Prevalence and risk potential of total serum vitamin C reduction

As shown in Figure 1(Fig. 1), when classified as adequate (23-85 µmol/L), insufficiency (11-22 µmol/L), and deficiency (<11µmol/L), the levels of total serum vitamin C revealed a different prevalence in each group of lifestyle behaviors. In this study, the prevalence of insufficiency and deficiency in group I (reference) was correlated with the previous findings in Thailand and other global regions (Pincemail et al., 2011[42]; Assantachai and Lekhakula, 2005[4]; Cahill et al., 2009[7]). For drinkers (Group II), the prevalence of insufficiency in our finding increased 1.4 times higher, as reported among drinkers in India, than the general population (Ravindran et al., 2011[44]). However, the same Indian report revealed a much higher vitamin C deficiency among drinkers (Ravindran et al., 2011[44]). Interestingly, the smokers (Group IV) showed the same deficiency prevalence as the outdoor workers (Group III), whereas the insufficiency prevalence of serum vitamin C of this group increased. These findings indicate that some degree of vitamin C reduction may be affected by other variables in our study group of smokers. 

To validate the reduction of the vitamin C levels, statistics and odds ratio were analyzed. The total serum vitamin C data were re-classified into two categories: adequate (23-85 µmol/L) and inadequate < 23 µmol/L (Figure 1(Fig. 1)). The odds ratio (OR) without (crude OR) and with an adjusted OR of the participants' education levels and genders were compared for the various lifestyle behaviors (Table 6(Tab. 6)). The ORs of the smokers and combined groups were statistically significant. However, when controlling for gender and education, the ORs markedly decreased but remained statistically significant. These findings suggest that smoking reduces vitamin C levels in humans. Our result correlated with those of other studies (Lykkesfeldt et al., 2000[32]; Dietrich et al., 2003[14]; Wei et al., 2001[58]).

### Dietary intake and vitamin C level

It is well known that humans rely on exogenous sources of vitamin C. This study assessed dietary intake through the 24-hour dietary recall method. The vitamin C dietary intake (regardless of sex) in this study was lower than Thai DRI. However, our results were correlated with the studies by Ravindran et al. in 2011 and Rojroongwasinkul et al. in 2013, who reported low vitamin C dietary intakes among the elderly Indian population and Thai children (Ravindran et al., 2011[44]; Rojroongwasinkul et al., 2013[46]). In addition, a low vitamin C dietary intake among non-smoker and smoker was also reported (Jitnarin et al., 2014[24]). The low vitamin C dietary intake might be caused by the low educational level and also low income (Shohaimi et al., 2004[50]; Mosdøl et al., 2008[35]). Regarding the correlation between total serum vitamin C and vitamin C dietary intake, a correlation of 0.402 was only observed in group I, with statistical significance. This correlation was similar to the correlation between plasma vitamin C and vitamin C dietary intake using the 24-hour dietary recall method (0.46), in the population survey, which ranged from one to twelve days (Dehghan et al., 2007[11]). In addition, a low correlation (r = 0.2) between plasma vitamin C and vitamin C dietary intake was also observed by another report group (Ravindran et al., 2011[44]). However, the other four groups in this study did not show a correlation between vitamin C dietary intake and corresponding serum vitamin C levels. The low and no correlation between serum vitamin C and dietary intake might be affected by food processing and storage, bioavailability, and error of dietary recall from participants (Dehghan et al., 2007[11]). In addition, the high level of free radicals generated from cigarette smoking leads to high usage of vitamin C and might be involved (Eiserich et al., 1995[16]; Pelletier, 1997[39]; Alberg, 2002[1]). Moreover, it might be related to half-life of vitamin C in the body pool, which is approximately 16 days (Yung et al., 1978[61]; Hellman and Burn, 1958[21]). Awareness and knowledge of selecting the appropriate food to consume is an important factor to maintain adequate level of vitamin C in people.

## Conclusions

In this cross-sectional study, we investigated the total serum vitamin C level and vitamin C dietary intake across various lifestyle behaviors of Thai adults. The reference group (Group I) had the highest total serum vitamin C level compared to the other four groups. The lowest total serum vitamin C belonged to Group V (combined). The prevalence of total serum vitamin C deficiency (< 11 µmol/L) was demonstrated in the following order: Group V (combined) > III (outdoor workers) > IV (smokers) > II (drinkers) > I (reference). Vitamin C insufficiency (11–22 µmol/L) was demonstrated in the following order: Group IV (smokers) > V (combined) > II (drinkers) > III (outdoor workers) > I (reference). The likelihood of having an inadequate total serum vitamin C (< 23µmol/L) was observed in the following order: Group V (combined) > IV (smokers) > III (outdoor workers) > II (drinkers) when compared to Group I (reference). The vitamin C dietary intakes across the five various lifestyle behaviors were similar but lower than the Thai DRI. Total serum vitamin C was positively correlated with vitamin C dietary intake for only the reference group. In summary, the total serum vitamin C level in this study can be used as preliminary data to understand the risk of vitamin C deficiency for various lifestyle behaviors among cigarette smokers, alcohol drinkers and outdoor workers. 

## Acknowledgements

We are thankful to Dr. Somsak Wongsawass, the ASEAN Institute for Health Development, Mahidol University, for his statistical analysis guidance. This project was supported by House Osotspa Foods Co., Ltd., Thailand and House Foods Group Inc., Japan. It was also partially supported by Mahidol University and the Office of the Higher Education Commission, Mahidol University, under the National Research Universities Initiative and Annual Government Grant under Mahidol University (2558-2560 B.E.).

## Conflicts of interests

The authors declare that they have no conflict of interest.

## Supplementary Material

Supplementary data

## Figures and Tables

**Table 1:**
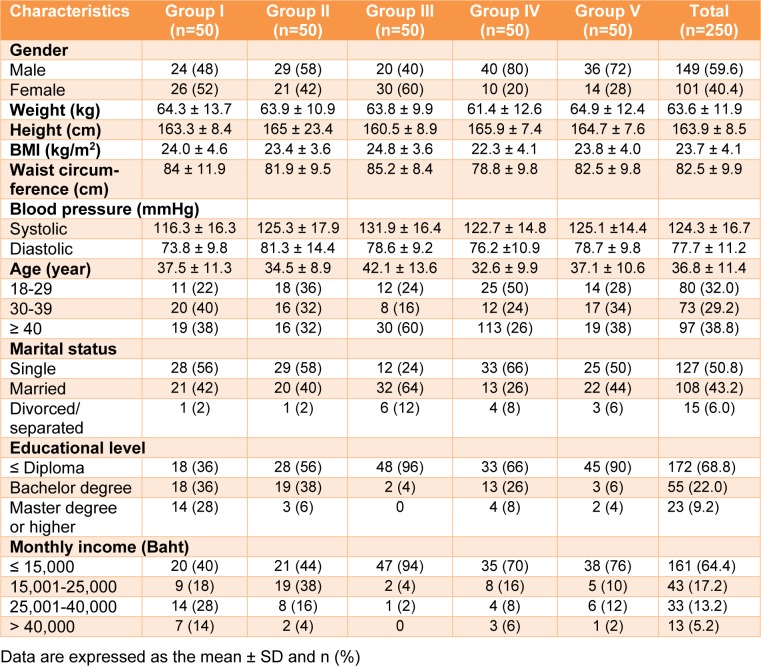
Sociodemographic and anthropometric profiles of the participants

**Table 2:**
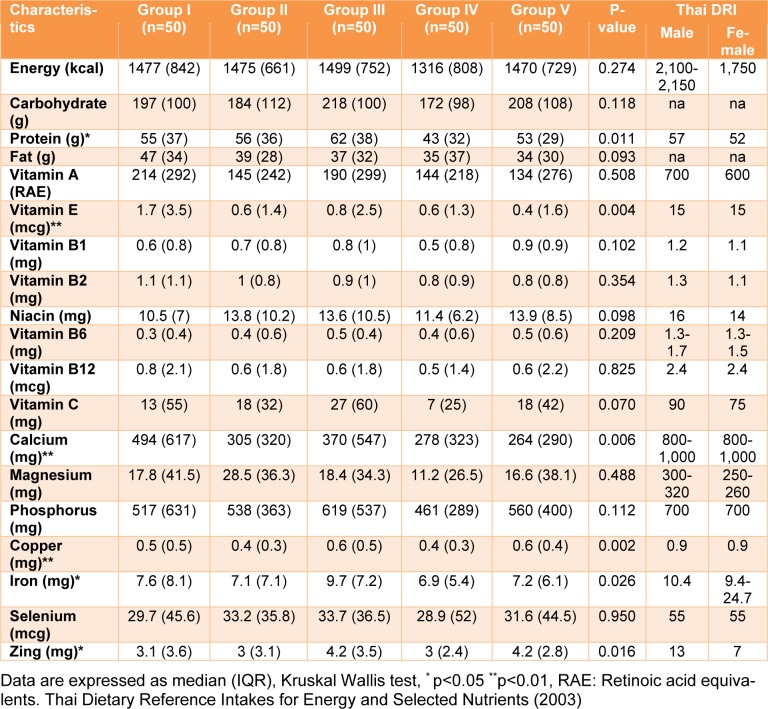
Nutrient intakes of the various lifestyle behavior groups

**Table 3:**
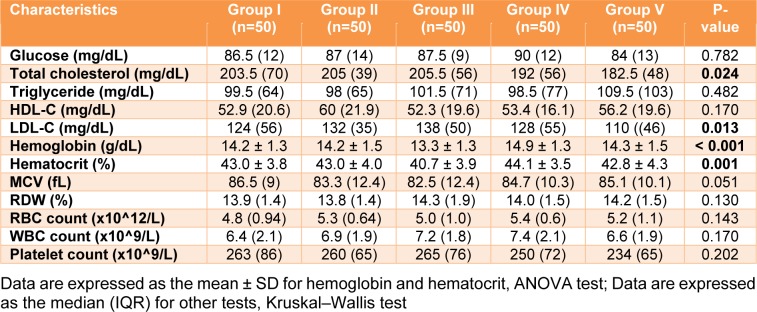
Baseline levels of glucose, lipid profiles, and complete blood counts

**Table 4:**
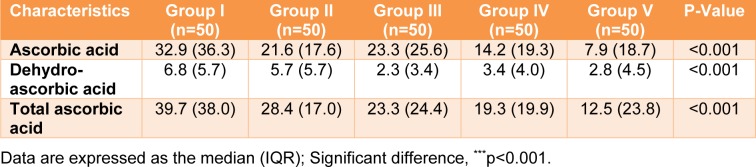
Serum ascorbic acid, dehydroascorbic acid, and total ascorbic acid (µmol/L) in various lifestyle behavior groups

**Table 5:**
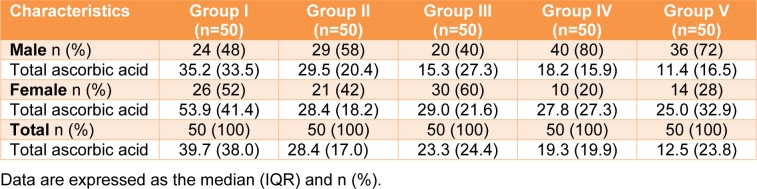
Total serum ascorbic acid (µmol/L) stratified by gender

**Table 6:**
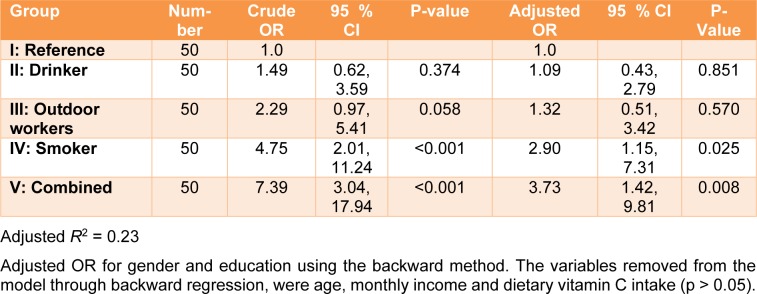
Crude and adjusted odds ratio of inadequate total serum vitamin C (<23 µmol/L) in relation to various lifestyle behaviors

**Table 7:**
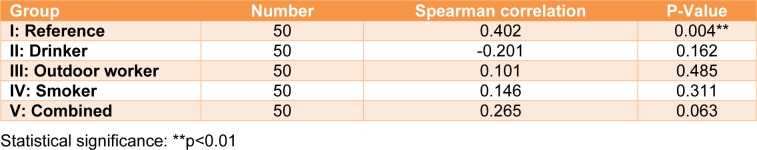
Spearman's correlation for total serum vitamin C and vitamin C dietary intake
