# Supplementary material for: Lifestyle behaviors and serum vitamin C in the Thai population in Bangkok Metropolitan
Source: EXCLI J. 2018 May 16;17:452–66. doi: 10.17179/excli2018-1203 (PMC6046627; doi:10.17179/excli2018-1203)
Supplement: Supplementary data [file EXCLI-17-452-s-001.pdf]

EXCLI Journal 2018;17:452-466 – ISSN 1611-2156
Received: March 14, 2018, accepted: April 17, 2018, published: May 16, 2018

Supplementary data to:

LIFESTYLE BEHAVIORS AND SERUM VITAMIN C IN THE THAI POPULATION IN BANGKOK METROPOLITAN

Somchai Boonpangrak[1,*], Tanawut Tantimongcolwat[1], Lertyot Treeratanapiboon[2], Pairoj Leelahakul[3], Virapong Prachayasittikul[4]

[1] Center for Research and Innovation, Faculty of Medical Technology, Mahidol University, Bangkok 73170, Thailand
[2] Department of Community Medical Technology, Faculty of Medical Technology, Mahidol University, Bangkok 73170, Thailand
[3] Department of Clinical Chemistry, Faculty of Medical Technology, Mahidol University, Bangkok 73170, Thailand
[4] Department of Clinical Microbiology and Applied Technology, Faculty of Medical Technology, Mahidol University, Bangkok 73170, Thailand

***** Corresponding author: E-mail: somchai.boo@mahidol.ac.th, Phone: +66 2 441 4374, Fax: +66 2 441 4380

http://dx.doi.org/10.17179/excli2018-1203

This is an Open Access article distributed under the terms of the Creative Commons Attribution License (http://creativecommons.org/licenses/by/4.0/).

Participant no.	Group	Sex	Age (Year)	Marital Status	Education	Income (Baht)	AA (mg/dL)	DHAA (mg/dL)	Total AA (mg/dL)	Dietary vitamin C intake (mg)	Total AA group
1	1	2	49	2	3	5	0.97	0.22	1.19	9.78	0.00
2	1	2	41	2	1	2	0.94	0.16	1.10	71.57	0.00
3	1	1	50	2	1	2	1.07	0.26	1.33	296.27	0.00
4	1	2	55	2	2	5	1.00	0.13	1.13	15.82	0.00
5	1	2	51	1	2	4	1.28	0.25	1.53	63.27	0.00
6	1	2	37	2	3	5	0.93	0.09	1.02	153.19	0.00
7	1	1	50	2	3	5	0.63	0.27	0.90	4.83	0.00
8	1	2	38	1	3	3	0.48	0.07	0.55	0.00	0.00
9	1	2	34	2	2	3	0.41	0.26	0.67	625.56	0.00
10	1	2	33	1	1	3	0.93	0.15	1.08	279.42	0.00
11	1	1	31	1	2	3	0.03	0.09	0.12	1.77	1.00
12	1	1	32	1	2	3	0.57	0.11	0.68	0.02	0.00
13	1	2	37	2	1	1	0.13	0.07	0.20	4.50	1.00
14	1	2	38	2	1	1	0.00	0.04	0.04	39.20	1.00
15	1	2	19	1	1	1	0.20	0.30	0.50	3.46	0.00
16	1	2	32	2	1	1	0.22	0.08	0.30	0.00	1.00
17	1	1	31	3	1	2	0.53	0.00	0.53	0.00	0.00
18	1	1	48	2	1	2	0.31	0.05	0.36	38.86	1.00
19	1	1	43	2	1	2	0.88	0.03	0.91	0.00	0.00
20	1	1	49	2	1	4	0.87	0.01	0.88	3.74	0.00
21	1	2	55	2	1	1	0.64	0.04	0.68	0.00	0.00
22	1	2	37	2	1	1	0.57	0.09	0.66	10.20	0.00
23	1	2	59	1	1	1	0.14	0.11	0.25	7.06	1.00
24	1	2	49	2	1	1	0.42	0.13	0.55	0.00	0.00
25	1	2	19	1	1	1	0.36	0.07	0.43	0.00	0.00
26	1	2	58	2	1	1	0.12	0.15	0.27	3.81	1.00
27	1	2	21	1	2	1	0.63	0.32	0.95	26.19	0.00
28	1	2	21	1	2	1	0.70	0.24	0.94	26.34	0.00
29	1	2	21	1	2	1	1.53	0.16	1.69	100.64	0.00
30	1	2	21	1	2	1	1.75	-0.10	1.65	75.60	0.00
31	1	2	27	1	2	4	1.30	0.13	1.43	9.82	0.00
32	1	1	41	2	2	4	1.58	0.38	1.96	126.87	0.00
33	1	1	32	1	1	2	0.52	0.19	0.71	1.00	0.00
34	1	1	23	1	2	3	0.77	0.22	0.99	149.53	0.00
35	1	1	35	1	3	4	1.04	0.12	1.16	0.00	0.00
36	1	1	37	1	3	4	0.23	0.03	0.26	1.53	1.00
37	1	1	23	1	2	3	0.10	0.06	0.16	45.14	1.00
38	1	1	35	1	2	4	0.20	0.10	0.30	40.43	1.00

39	1	2	44	2	3	5	1.26	0.14	1.40	6.50	0.00
40	1	1	36	1	3	4	0.71	0.10	0.81	81.83	0.00
41	1	2	46	1	3	4	1.19	0.08	1.27	151.43	0.00
42	1	1	47	2	2	4	0.77	0.15	0.92	37.96	0.00
43	1	1	25	1	3	3	0.44	0.08	0.52	56.20	0.00
44	1	1	34	2	3	5	0.17	0.12	0.29	9.37	1.00
45	1	1	30	1	2	3	0.05	0.05	0.10	27.79	1.00
46	1	2	62	1	3	5	0.86	0.14	1.00	51.67	0.00
47	1	1	31	1	3	4	0.35	0.14	0.49	28.38	0.00
48	1	1	34	1	3	4	0.58	0.22	0.80	48.42	0.00
49	1	1	29	1	2	4	0.33	0.12	0.45	7.43	0.00
50	1	1	46	1	2	4	0.49	0.07	0.56	5.32	0.00
51	2	2	40	1	2	4	0.79	0.16	0.95	0.00	0.00
52	2	1	31	1	2	3	1.11	0.10	1.21	19.97	0.00
53	2	1	42	2	1	1	0.05	0.04	0.09	43.85	1.00
54	2	2	42	1	1	3	0.28	0.07	0.35	9.00	1.00
55	2	1	26	1	2	1	0.34	0.11	0.45	6.96	0.00
56	2	1	49	2	1	2	0.24	0.04	0.28	29.02	1.00
57	2	1	40	1	1	1	0.03	0.05	0.08	23.97	1.00
58	2	1	26	2	1	2	0.56	-0.04	0.52	6.50	0.00
59	2	1	53	2	1	5	0.55	0.06	0.61	20.79	0.00
60	2	2	33	2	1	1	0.32	0.05	0.37	28.40	1.00
61	2	2	44	2	1	1	0.76	0.20	0.96	0.00	0.00
62	2	2	22	1	2	1	0.41	0.24	0.65	15.48	0.00
63	2	1	54	2	1	3	0.67	0.08	0.75	0.00	0.00
64	2	1	43	2	1	3	0.27	0.14	0.41	0.00	0.00
65	2	1	43	1	1	2	0.87	0.02	0.89	55.16	0.00
66	2	2	56	2	2	3	0.87	0.06	0.93	18.65	0.00
67	2	1	29	1	2	2	0.30	0.26	0.56	34.93	0.00
68	2	2	32	1	2	3	0.88	0.19	1.07	48.34	0.00
69	2	1	31	2	1	3	0.43	0.25	0.68	9.19	0.00
70	2	1	23	1	2	3	0.36	0.14	0.50	52.21	0.00
71	2	1	34	1	3	4	0.61	0.01	0.62	17.53	0.00
72	2	1	32	1	1	2	0.74	0.00	0.74	0.00	0.00
73	2	1	35	1	1	3	0.50	0.10	0.60	24.47	0.00
74	2	1	48	2	1	4	0.68	0.00	0.68	18.46	0.00
75	2	1	29	1	2	3	0.62	0.05	0.67	1.47	0.00
76	2	1	26	1	3	4	0.35	0.10	0.45	23.40	0.00
77	2	1	40	1	2	3	0.45	0.05	0.50	15.20	0.00
78	2	1	23	1	2	3	0.64	0.09	0.73	1.59	0.00
79	2	1	47	2	1	3	0.23	0.09	0.32	7.69	1.00
80	2	1	29	1	2	4	0.18	0.07	0.25	10.79	1.00
81	2	1	36	1	2	4	0.13	0.08	0.21	48.75	1.00

82	2	2	27	1	2	3	0.37	0.29	0.66	52.24	0.00
83	2	1	27	1	2	1	0.17	0.07	0.24	2.96	1.00
84	2	2	22	1	1	2	0.57	0.10	0.67	0.00	0.00
85	2	2	33	1	1	2	0.31	0.04	0.35	9.00	1.00
86	2	1	38	2	1	2	0.54	0.12	0.66	27.41	0.00
87	2	2	37	2	2	1	0.53	0.14	0.67	16.80	0.00
88	2	1	40	2	2	4	0.32	0.10	0.42	1.62	0.00
89	2	2	35	1	3	5	0.34	0.10	0.44	107.65	0.00
90	2	2	44	1	1	3	0.37	0.13	0.50	20.52	0.00
91	2	1	36	2	2	2	0.51	0.13	0.64	70.88	0.00
92	2	2	25	2	1	3	0.41	0.09	0.50	70.75	0.00
93	2	2	33	2	1	2	0.38	0.12	0.50	0.00	0.00
94	2	2	27	1	1	3	0.19	0.09	0.28	29.57	1.00
95	2	2	28	1	1	2	0.03	0.13	0.16	413.27	1.00
96	2	1	30	2	2	3	0.26	0.06	0.31	16.80	1.00
97	2	2	27	2	1	2	0.42	0.08	0.50	57.80	0.00
98	2	2	21	3	1	2	0.07	0.08	0.15	117.82	1.00
99	2	2	28	1	1	3	0.08	0.06	0.14	0.00	1.00
100	2	2	31	1	1	4	0.32	0.05	0.37	6.62	1.00
101	3	2	55	3	1	1	0.86	-0.01	0.85	44.45	0.00
102	3	2	48	2	1	1	0.59	0.04	0.63	6.15	0.00
103	3	2	50	2	1	1	0.08	0.01	0.09	111.36	1.00
104	3	2	67	2	1	2	0.11	0.06	0.17	7.84	1.00
105	3	1	28	1	1	2	0.57	0.01	0.58	118.28	0.00
106	3	2	55	3	1	1	0.70	0.07	0.77	20.97	0.00
107	3	2	64	2	1	1	0.93	-0.02	0.91	11.21	0.00
108	3	2	32	2	1	1	0.29	0.03	0.32	3.90	1.00
109	3	2	25	1	1	1	0.07	0.06	0.13	11.80	1.00
110	3	2	53	3	1	2	0.28	0.03	0.31	96.74	1.00
111	3	2	60	1	1	1	0.73	0.02	0.75	20.16	0.00
112	3	2	48	2	1	1	0.27	0.11	0.38	1.41	1.00
113	3	1	32	1	1	1	0.20	0.05	0.25	65.37	1.00
114	3	2	55	2	1	1	0.63	0.10	0.73	21.18	0.00
115	3	2	55	2	1	1	0.52	0.00	0.52	83.17	0.00
116	3	2	56	2	1	1	0.45	-0.05	0.40	4.50	0.00
117	3	2	19	2	1	2	0.49	0.01	0.50	46.36	0.00
118	3	2	22	2	1	1	0.31	0.04	0.35	83.99	1.00
119	3	1	36	2	1	1	0.41	0.00	0.41	93.83	0.00
120	3	2	57	2	1	1	0.90	0.06	0.96	70.28	0.00
121	3	1	42	3	1	1	0.18	0.00	0.18	13.21	1.00
122	3	1	18	1	1	1	0.89	0.02	0.91	0.00	0.00
123	3	2	20	2	1	2	0.08	0.01	0.09	44.10	1.00
124	3	2	25	2	1	2	0.40	0.00	0.40	4.11	0.00

125	3	2	20	1	1	1	0.61	0.05	0.66	9.57	0.00
126	3	1	32	2	1	2	0.07	0.04	0.11	75.51	1.00
127	3	1	50	2	1	2	0.14	0.03	0.17	0.00	1.00
128	3	2	43	2	1	1	0.64	0.01	0.65	45.43	0.00
129	3	1	51	2	1	2	0.63	0.11	0.74	47.83	0.00
130	3	1	28	1	1	1	0.07	0.09	0.16	65.62	1.00
131	3	1	27	2	1	2	0.02	0.13	0.15	2.74	1.00
132	3	1	42	2	1	1	0.24	0.10	0.34	55.34	1.00
133	3	2	51	2	1	1	0.50	0.13	0.63	197.07	0.00
134	3	2	47	2	1	2	0.70	0.02	0.72	8.50	0.00
135	3	2	38	2	1	2	0.32	0.15	0.47	79.12	0.00
136	3	2	45	3	1	2	0.71	0.03	0.74	59.96	0.00
137	3	1	62	2	1	2	0.03	0.02	0.05	55.74	1.00
138	3	1	67	1	1	2	0.37	0.04	0.41	0.00	0.00
139	3	2	36	2	1	2	0.51	-0.03	0.48	6.00	0.00
140	3	2	47	2	1	1	0.53	-0.02	0.51	63.59	0.00
141	3	1	48	2	1	2	0.03	0.06	0.09	0.00	1.00
142	3	2	40	2	1	1	0.39	0.02	0.41	4.94	0.00
143	3	1	53	2	1	2	0.28	0.01	0.29	1.47	1.00
144	3	1	46	2	2	4	0.57	0.11	0.68	241.98	0.00
145	3	1	48	1	1	2	0.12	0.13	0.25	48.01	1.00
146	3	1	49	3	1	3	0.92	0.04	0.96	40.03	0.00
147	3	1	35	2	2	2	0.06	0.02	0.08	5.49	1.00
148	3	2	34	1	1	3	0.22	0.04	0.26	1.27	1.00
149	3	2	20	1	1	2	0.54	0.11	0.65	32.28	0.00
150	3	1	26	1	1	1	0.58	0.07	0.65	5.15	0.00
151	4	1	32	2	1	2	0.00	0.01	0.01	22.26	1.00
152	4	1	40	3	1	2	0.05	0.04	0.09	1.84	1.00
153	4	1	37	2	1	2	0.06	0.08	0.14	0.00	1.00
154	4	1	26	2	1	2	1.27	0.19	1.46	0.00	0.00
155	4	1	58	1	1	3	0.01	0.05	0.06	3.48	1.00
156	4	1	51	2	1	3	0.25	0.06	0.31	22.10	1.00
157	4	1	27	1	2	2	0.42	0.03	0.45	0.00	0.00
158	4	1	35	1	2	2	0.95	-0.02	0.93	102.50	0.00
159	4	2	47	1	3	4	1.08	-0.08	1.00	6.94	0.00
160	4	1	26	1	2	3	0.62	0.14	0.76	2.00	0.00
161	4	1	24	1	2	3	0.54	0.02	0.56	21.94	0.00
162	4	2	38	1	3	5	0.42	0.13	0.55	3.72	0.00
163	4	2	32	3	2	5	0.09	0.05	0.14	42.89	1.00
164	4	1	37	1	3	4	0.08	0.06	0.14	2.25	1.00
165	4	1	20	1	1	2	0.18	0.08	0.26	6.79	1.00
166	4	1	30	1	2	3	0.41	0.05	0.46	62.56	0.00
167	4	1	21	1	1	1	0.21	0.10	0.31	570.00	1.00

168	4	1	26	1	1	2	0.10	0.16	0.26	73.15	1.00
169	4	1	43	2	2	4	0.13	0.17	0.33	0.00	1.00
170	4	1	41	1	1	3	0.24	0.11	0.35	0.00	1.00
171	4	1	21	1	1	2	0.48	0.10	0.58	51.15	0.00
172	4	1	24	1	2	2	0.40	0.09	0.49	0.00	0.00
173	4	2	44	2	1	2	0.54	0.06	0.60	109.12	0.00
174	4	1	40	2	3	4	0.17	0.05	0.22	12.32	1.00
175	4	2	28	2	1	2	0.07	0.13	0.20	0.00	1.00
176	4	2	25	1	2	2	0.46	0.10	0.56	4.91	0.00
177	4	1	26	1	1	3	0.73	0.02	0.75	207.87	0.00
178	4	1	49	1	2	5	0.12	0.03	0.15	0.00	1.00
179	4	1	23	1	1	2	0.17	0.03	0.20	2.04	1.00
180	4	1	22	1	1	2	0.17	0.05	0.22	0.36	1.00
181	4	2	24	1	2	2	0.14	0.06	0.20	0.23	1.00
182	4	1	35	1	1	2	0.34	0.09	0.43	0.00	0.00
183	4	1	29	1	2	2	0.87	0.10	0.97	5.19	0.00
184	4	1	33	2	1	1	0.28	0.13	0.41	12.82	0.00
185	4	1	26	1	1	2	0.29	0.07	0.36	8.52	1.00
186	4	2	53	2	1	1	0.86	0.04	0.90	3.19	0.00
187	4	2	40	2	1	1	0.05	0.19	0.24	0.00	1.00
188	4	2	53	3	1	1	0.28	0.14	0.42	15.90	0.00
189	4	1	49	1	1	3	0.16	0.04	0.20	0.07	1.00
190	4	1	24	1	1	2	0.44	0.02	0.46	0.00	0.00
191	4	1	33	2	1	1	0.12	0.03	0.15	1.53	1.00
192	4	1	24	1	2	2	0.26	0.03	0.29	18.91	1.00
193	4	1	27	3	1	2	0.56	-0.01	0.55	34.17	0.00
194	4	1	27	1	1	2	0.07	0.05	0.12	17.43	1.00
195	4	1	24	1	1	2	0.09	0.11	0.20	8.80	1.00
196	4	1	30	1	1	2	0.11	0.06	0.17	6.92	1.00
197	4	1	32	1	1	1	0.29	0.07	0.36	18.10	1.00
198	4	1	25	1	1	2	0.28	0.06	0.34	44.05	1.00
199	4	1	28	2	1	2	0.24	0.15	0.39	53.30	1.00
200	4	1	22	1	1	2	0.04	0.07	0.11	32.66	1.00
201	5	1	49	2	1	1	0.08	0.04	0.12	5.67	1.00
202	5	2	49	3	1	1	0.75	0.03	0.78	171.98	0.00
203	5	1	32	1	1	1	0.12	0.05	0.17	43.68	1.00
204	5	1	25	2	1	2	0.14	0.03	0.17	41.39	1.00
205	5	1	60	2	1	2	0.04	0.01	0.05	5.85	1.00
206	5	1	48	2	1	2	0.06	0.02	0.08	250.19	1.00
207	5	1	30	2	1	2	0.03	0.04	0.07	0.00	1.00
208	5	1	54	2	1	1	0.08	0.03	0.11	74.81	1.00
209	5	2	35	2	1	1	0.00	0.02	0.02	55.63	1.00
210	5	2	42	2	1	1	0.13	0.02	0.15	9.68	1.00

211	5	1	27	1	1	2	0.00	0.01	0.01	13.37	1.00
212	5	1	30	1	1	2	0.10	0.04	0.14	0.00	1.00
213	5	1	34	2	1	2	0.14	0.08	0.22	13.42	1.00
214	5	1	32	1	1	2	0.00	0.05	0.05	5.75	1.00
215	5	1	53	2	1	2	0.46	0.06	0.52	58.06	0.00
216	5	1	39	2	1	2	0.50	0.14	0.64	4.66	0.00
217	5	2	48	2	1	1	0.12	0.10	0.22	3.15	1.00
218	5	1	37	2	1	1	0.15	0.10	0.25	21.99	1.00
219	5	1	51	2	1	1	0.00	0.02	0.02	0.00	1.00
220	5	1	36	2	1	2	0.24	0.02	0.26	3.99	1.00
221	5	2	52	3	1	1	0.98	-0.04	0.94	46.62	0.00
222	5	1	63	2	1	1	0.02	0.02	0.04	40.00	1.00
223	5	1	55	2	1	3	0.55	0.00	0.55	0.00	0.00
224	5	2	39	2	1	1	0.27	0.03	0.30	92.78	1.00
225	5	1	27	1	1	2	0.00	0.01	0.01	4.94	1.00
226	5	1	21	1	1	2	0.12	0.02	0.14	11.87	1.00
227	5	1	41	1	1	1	0.25	0.11	0.36	29.22	1.00
228	5	1	24	1	1	2	0.35	0.24	0.59	47.18	0.00
229	5	1	29	1	1	2	0.12	0.08	0.20	0.00	1.00
230	5	2	31	1	2	4	0.39	0.11	0.50	60.97	0.00
231	5	2	29	1	3	4	0.86	0.16	1.02	23.25	0.00
232	5	1	29	1	3	4	0.27	0.04	0.31	8.61	1.00
233	5	1	41	1	1	3	0.06	0.03	0.09	25.20	1.00
234	5	2	37	1	1	4	0.33	0.19	0.52	19.27	0.00
235	5	1	30	1	1	2	0.07	0.04	0.11	3.18	1.00
236	5	1	30	1	1	2	0.13	0.05	0.18	0.00	1.00
237	5	2	44	2	1	2	0.33	0.05	0.38	12.63	1.00
238	5	1	39	1	1	5	0.17	0.10	0.27	1,193.50	1.00
239	5	1	27	1	1	3	0.17	0.07	0.24	0.39	1.00
240	5	1	18	1	1	1	0.66	0.06	0.72	44.89	0.00
241	5	1	29	1	1	2	0.46	0.10	0.56	1.13	0.00
242	5	1	41	3	1	3	0.11	0.10	0.21	16.80	1.00
243	5	1	38	2	2	2	0.26	0.13	0.39	0.00	1.00
244	5	1	42	1	1	2	0.44	0.12	0.56	52.31	0.00
245	5	1	25	1	2	4	0.47	0.12	0.59	106.71	0.00
246	5	2	40	2	1	3	0.02	0.05	0.07	25.89	1.00
247	5	2	32	1	1	2	0.65	0.06	0.71	21.62	0.00
248	5	1	41	2	1	2	0.09	0.10	0.19	38.65	1.00
249	5	2	27	1	1	4	0.61	0.07	0.68	65.39	0.00
250	5	2	23	1	1	2	0.13	0.02	0.15	13.23	1.00

Abbreviations:

AA = Ascorbic acid

DHAA= Dehydroascorbic acid

Total AA = Total ascorbic acid

mg= milligram

mg/dL=milligram/deciliter

Code identifiers:

Group: 1= Reference 2= Drinker 3= Outdoor worker 4= Smoker 5= Combined group

Sex: 1= Male 2= Female

Marital status: 1= Single 2= Married 3= Divorced/Separated

Education: 1≤ Diploma 2= Bachelor degree 3= Master degree or higher

Income: 1 ≤9,000 2= 9,000-15,000 3= 15,001-25,000 4= 25,001-40,000

Total ascorbic acid group: 1= Adequate ≥ 0.4 mg/dL (≥ 23 micromole/L)   2= Inadequate < 0.4 mg/dL (< 23 micromole/L)
